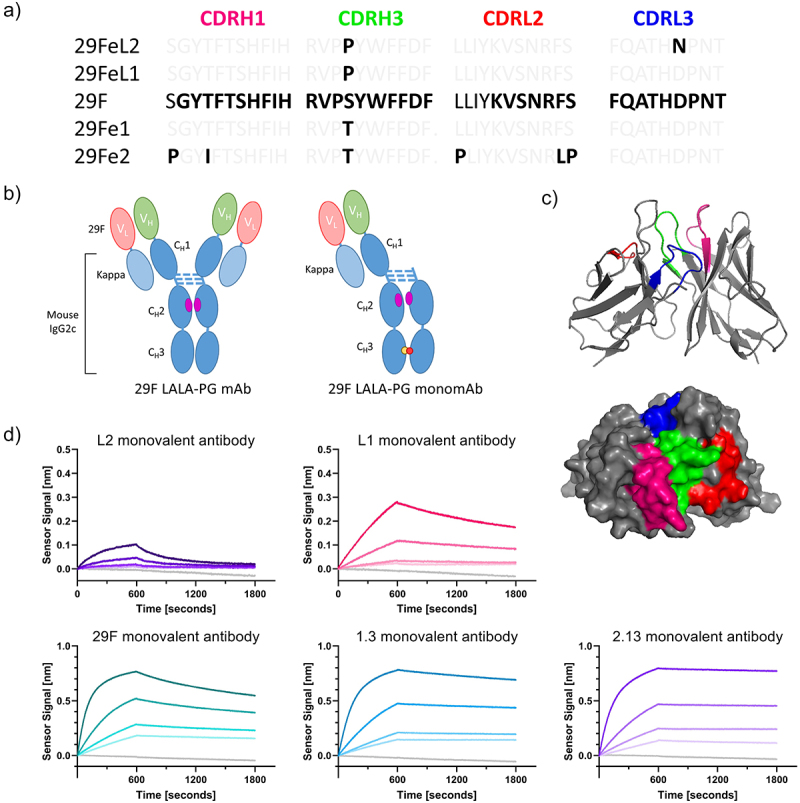# Correction

**DOI:** 10.1080/19420862.2024.2364972

**Published:** 2024-06-07

## Article title: An affinity threshold for maximum efficacy in anti-PD-1 immunotherapy

**Authors**: Sarah C. Cowles, Allison Sheen, Luciano Santollani, Emi A. Lutz, Brianna M. Lax, Joseph R. Palmeri, Gordon J. Freeman, K. Dane Wittrup

**Journal**: mAbs

**DOI**: https://doi.org/10.1080/19420862.2022.2088454

It has been noted by the authors that Figure 1a was published with an error. Sequencing confirmed that the antibody CDRH1 for all the proteins produced for this work is GYTFTSHFIH, with the exception of the highest affinity mutant, as noted in Figure 1a. This corrected CDRH1 sequence is the same as the original 29F.1A12 sequence discovered in the Freeman Lab. The correction has not changed the interpretation or original conclusions of the article. The authors apologize for any inconvenience caused.

**NEW Figure 1:**